# Neuroblastoma and pre-B lymphoma cells share expression of key transcription factors but display tissue restricted target gene expression

**DOI:** 10.1186/1471-2407-4-80

**Published:** 2004-11-15

**Authors:** Anna Lagergren, Christina Manetopoulos, Håkan Axelson, Mikael Sigvardsson

**Affiliations:** 1 Stem Cell Center, Lund University, S-221 85 Lund, Sweden; 2 Department of Laboratory Medicine, Division of Molecular Medicine, Lund University, University Hospital MAS, S-205 02 Malmö, Sweden

## Abstract

**Background:**

Transcription factors are frequently involved in the process of cellular transformation, and many malignancies are characterized by a distinct genetic event affecting a specific transcription factor. This probably reflects a tissue specific ability of transcription factors to contribute to the generation of cancer but very little is known about the precise mechanisms that governs these restricted effects.

**Methods:**

To investigate this selectivity in target gene activation we compared the overall gene expression patterns by micro-array analysis and expression of target genes for the transcription factor EBF in lymphoma and neuroblastoma cells by RT-PCR. The presence of transcription factors in the different model cell lines was further investigated by EMSA analysis.

**Results:**

In pre-B cells mb-1 and CD19 are regulate by EBF-1 in collaboration with Pax-5 and E-proteins. We here show that neuroblastoma cells express these three, for B cell development crucial transcription factors, but nevertheless fail to express detectable levels of their known target genes. Expression of mb-1 could, however, be induced in neuroblastoma cells after disruption of the chromatin structure by treatment with 5-azacytidine and Trichostatin A.

**Conclusion:**

These data suggest that transcription factors are able to selectively activate target genes in different tissues and that chromatin structure plays a key role in the regulation of this activity.

## Background

The complex process of tumor development often involves changes in the transcription regulatory networks. In human cancer, genetic changes involving the transcription factor *p53 *gene is particularly common and the gene is found mutated in cancers originating from numerous cell types. This factor is, however, broadly expressed and directly involved in cell cycle regulation and apoptosis explaining the common involvement of the protein in tumor development. Many malignancies are characterized by specific chromosomal translocations that frequently affects the expression or structure of transcription factors with a more tissue specific expression pattern, often with an important function during development [1]. Examples of this can be found within the hematopoetic system where for instance translocations of *Tal-1 *is associated with T cell leukemia's [2] while modified *BCL-6 *[3] or *c-myc *[4] is associated with B cell non-Hodgkin's lymphomas. The close correlation with a specific tumor type and pathology to a specific transcription factor modification could well be explained by differential expression patterns and accessibility of the gene for translocation events. Another possibility could be that the action of the transcription factor is context dependent and therefore the ability of the modified protein to contribute to tumor development depends on the cell in which it arises.

To investigate mechanisms involved in lineage specific gene regulation and transcription factor target gene selection in tumor cells we have compared transcription factor expression in neuroblastoma and pre-B lymphoma cells. This revealed that both these highly divergent tumor types expressed the transcription factor EBF [5] that has been contributed a central role in B cell development [6]. The protein is a helix-loop-helix family member [7,8] essential for B-lymphopoesis in mice [6] where it has been shown to regulate a large number of pre-B cell restricted genes including the surrogate light chains [9], CD19 [10] and the signal transduction proteins Igα (*mb-1*) [7,11] and Igβ (*B29*) [12]. The EBF-1 protein is highly conserved between human and mouse [13] and it also appears as if the target gene spectra has been conserved between species even though the primary promoter sequences of these genes has diverged [13,14]. Pre-B cells express exclusively EBF-1 [15] while neuroblastoma cells express other family members including EBF-2 and -3 [5]. There are limited information about EBF target genes in neuroblastoma cells but binding sites for EBF proteins were identified in the promoters controlling the expression of the neuron restricted *Chromogranin A *(*CGA*) and *SCG10 *genes [5].

In the present study we were interested in the mechanisms of tissue specific target gene activation by comparing transcription factor function and gene expression patterns in neuroblastomas and pre-B cells. We here report that even though neuroblastoma cells express both Pax-5 and E-proteins, both suggested to be crucial co-activators for EBF target genes in pre-B cells, the cells do not express the pre-B cell restricted target genes *mb-1 *and *CD19*. The expression of the *mb-1 *gene could, however, be activated by treatment of the neuroblastoma cells with chromatin disrupting agents. This suggests that chromatin structure is a key component in the regulation of transcription factor function, by restricting the accessibility of target genes, possibly contributing to the apparent link between a transcription factor and specific malignancies.

## Methods

### Cell culture

HeLa, THP-1, KM3 and Nalm6 cells were grown in RPMI 1640 medium supplemented with 7.5% fetal calf serum (FCS), 10 mM HEPES, 2 mM pyruvate and 50 μg/ml gentamicin (complete RPMI media) (Life Technologies) at 37°C and 5% CO_2_. SH-SY5Y, IMR-32 and SK-N-BE(2)c neuroblastoma cells were cultured in Eagle's Minimum Essential Medium (MEM) with 10% FCS, 100 U/ml penicillin and 100 μg/ml streptomycin in an atmosphere of 5% CO_2 _at 37°C (Life Technologies). KCN-69n and LA-N-1 neuroblastoma cells were cultured in RPMI 1640 medium supplemented with FCS and antibiotics as above. The SK-N-BE(2)c cells were differentiated with 10 μM all-*trans*-retinoic acid (RA) in MEM containing 10% FCS for 0, 2, 8, 24, and 96 hours. 5-azacytidine (5-azaC) treatments were performed on SK-N-BE(2)c and SH-SY5Y cells at a concentration of 400 nM. After 64 h, fresh medium containing 50 nM Trichostatin A (TSA) was added. The cells were harvested in PBS after 24 h.

### RT-PCR analysis

RNA was prepared from cells using Trizol (Life Technology) and cDNA was generated by annealing 1 μg of total RNA to 0,5 μg of random hexamers in 10 μl DEPC-treated water. Reverse transcriptase reactions were performed with 200 units of SuperScript Reverse Transcriptase (Life Technologies) in the manufacturers' buffer supplemented with 0,5 mM dNTP, 10 mM DTT and 20 units RNase inhibitor (Boeringer Mannheim, Bromma, Sweden) in a total volume of 20 μl, at 37°C for 1 hr. One-twentieth of the RT reactions were used in the PCR assays. PCR reactions were performed with 1 unit of Taq-polymerase (Life Technologies) in the manufacturers' buffer supplemented with 0.2 mM dNTP, in a total volume of 25 μl. GADPH was amplified by 25 cycles (94°C, 30 s, 55°C, 30 s and 72°C, 30 s) while 30 cycles were used to amplify CGA, SCG10, mb-1 and CD19 message (94°C, 30 s, 61°C, 30 s, 72°C 30 s). Primers were added to a final concentration of 1 mM. PCR products were blotted onto Hybond N^+ ^nylon membranes (Amersham) using capillary blotting with 0.4 M NaOH. Membranes were pre-hybridized in 5X Denhardt's, 6XSSC, 0.1% SDS and 50 μg/ml Salmon Sperm DNA, at 57°C for 90 minutes and hybridized with γ[^32^P] labeled oligonucleotide for 12 hours at 57°C in the same solution. Membranes were washed at room temperature 2 times in 2XSSC supplemented with 0.1% SDS for 15 minutes.

Oligonucleotides used for RT-PCR were:

*GADPH *sense; 5'-CCACCCATGGCAAATTCCATGGCA;

*GADPH *antisense; 5'-TCTAGACGGCAGGTCAGGTCCACC;

*CGA *sense: 5'-GAAGATGAACTCTCAGAGGTTC

*CGA *antisense: 5'-GGATCTCCTTGTAGCCAAGGCTCG

*CD19 *sense: 5'-AGTCATTGCTGAGCCTAGAGCTG

*CD19 *antisense: 5'-CTCGGAGTCCTCCTCACTGTCAG

*mb-1 *sense; 5'-CCAGCATCATTGATGGTGAGCC

*mb-1 *antisense: 5'-GACATCTCCTATGTTGAGGCTGC

*mb-1 *hybridization; 5'-CCCGCACAATAGCAGCAACAACGCCAACGT

*SCG10 *sense; 5'-ATGCTGTCACTGATCTGCTCTTGC

*SCG10 *antisense; 5'-CAGGTTGAACTGTCTGGCTGAAG

### EMSA

DNA probes were labeled with γ[^32^P] ATP by incubation with T4 polynucleotide kinase (Roche Molecular Biochemicals), annealed and purified on a 5% polyacrylamide Tris-borate-EDTA (TBE) gel. Nuclear extract [16], or *in vitro *transcribed-translated protein, was incubated with labeled probe (20,000 cpm, 3 fmol) for 30 min at room temperature in binding buffer (10 mM HEPES pH [7.9], 70 mM KCl, 1 mM Dithiothreitol, 1 mM EDTA, 2.5 mM MgCl_2_, 1 mM ZnCl_2_, 5% Glycerol) with 0.75 μg Poly(dI/dC) (Amersham Pharmacia). Antibodies (anti Pax-5 SC-1974, anti Pu.1 SC-352, anti actin SC-1616, ets1/2 SC-275 and anti myc SC-764 all from Santa Cruz Biotech and anti E2-2 from Pharmingen) were added 10 min before the addition of the DNA probe. The samples were separated on a 6% polyacrylamide TBE gel, which was dried and subjected to autoradiography.

Oligonucleotides used for EMSA were the following:

*mb-1 *sense 5'-AGCCACCTCTCAGGGGAATTGTGG;

*mb-1 *antisense 5'-CCACAATTCCCCTGAGAGGTGGCT;

*CD19*-BSAP sense 5'-GCAGACACCCATGGTTGAGTGCCCTCCAGG;

*CD19*-BSAP antisense 5'-CCTGGAGGGCACTCAACCATGGGTGTCTGC;

μ*E5 *sense: 5'-GGCCAGAACACCTGCAGACG;

μ*E5 *antisense: 5'-CGTCTGCAGGTGTTCTGGCC;

*Oct *binding site sense 5'-CATCTCAAGTGATTTGCATCGCATGAGACG;

*Oct *binding site antisense 5'-CGTCTCATGCGATGCAAATCACTTGAGATC;

*Lambda B *(Pu.1 site) sense: 5'-GAAAAAGAGAAATAAAAGGAAGTGAAACCA AG;

*Lambda B *antisense: 5'-CTTGGTTTCACTTCCTTTTATTTCTCTTTTTC;

*CRE *(ATF5 site) sense: TCA TGG TAA AAA TGA CGT CAT GGT AAT TA

*CRE *antisense: TAA TTA CCA TGA CGT CAT TTT TAC CAT GA

### cDNA micro-array analysis

RNA from Nalm6, SK-N-BE(2)c, SH-SY5Y, KM3, THP-1 and HeLa cells was extracted using Trizol™ (Invitrogen, Carlsbad, California). A common RNA control obtained by mixing a variety of cell lines was used for all hybridizations. RNA was concentrated to 50 μg total RNA (25 μg sample RNA and 25 μg control RNA) to generate aminoallyl-modified cDNA. Sample cDNA was labeled with Cy3-dCTP and control RNA was labeled with Cy5-dCTP using CyScribe Post-Labeling Kit (Amersham Pharmacia Biosciences). A hybridization solution was made by combining labeled cDNA with 20 μl Cot-1 DNA (1 mg/ml), 3 μl Poly dA (4 mg/ml) and 1.5 μl yeast t-RNA (4 mg/ml), dry down by speed-vac and resuspended in 40 μl Pronto! Universal Hybridization Solution™ (Pronto!™ Universal Microarray Reagent System, Corning). The hybridization solution was added to a pre-hybridized microarray slide (DNA microarrays were obtained from the SWEGENE DNA Microarray Resource Center, Lund University). The arrays were hybridized at 42°C for 18 hrs, washed according to the manufacturers recommendations (Pronto!™ Universal Microarray Reagent System, Corning), dried by centrifugation and scanned on Agilent microarray scanner. Scans were analyzed using GenePix Pro versions 4.0.1.9 and 4.1.1.4. BioArray Software Environment (BASE) (Saal et al. Genome Biology 2002, 3(8):software0003.1–0003.6). The settings for the analysis presented were, Background Correlation: Mean FG – Mean BG, Spot filter: (Raw) SNR ch1 mean > = 2, (Raw) SNR ch2 mean > = 2, (Raw) Flags = 0, (Raw) Spot diameter > = 40, Normalization: Lowess, Reporter filter: in # of assays = 16, Analysis: Hierarchical clustering (reporter)

## Results

### Neuroblastoma and lymphoma cell lines display similarities in overall gene expression patterns but not of known EBF target genes

**Figure 1:**
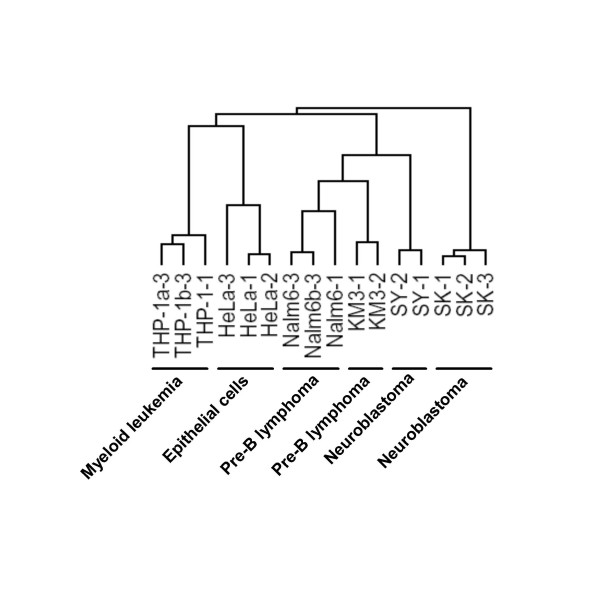
Pre-B cells and Neuroblastoma cells display similarities in overall gene expression patterns. The figure shows a hierarchical tree based on cDNA micro-array analysis of the expression of 2162 genes in a set of cell lines as indicated. Each individual hybridization experiment is represented by the name of the cell line and the number of the experiment. THP-1 is a human myeloma cell line, HeLa a human epithelial cell line, Nalm6 and KM3 represents human pre-B lymphoma. SY represents the neuroblastoma cell line SH-SY5Y and SK the neuroblastoma cell line SK-N-BE(2)c.

Knowing that neuroblastoma and pre-B cells share the expression of EBF [5,13,14], we wanted to investigate the potential similarities in overall gene expression patters in these two types of tumor cells. To this end we used cDNA micro-array analysis with material from two pre-B cell lines (Nalm6 and KM3), one EBF high and one EBF low expressing neuroblastoma cell line (SK-N-BE(2)c and SH-SY5Y) [5] (Data not shown, 13) one epithelial cell line (HeLa) and one myeloid cell line (THP-1). The level of specific mRNAs in the samples were compared to that obtained by a pooled reference RNA on cDNA gene chip (Appendix A). This allowed us to reliably investigate variations in expression of 2 162 genes in our cell lines and to group the different samples based on overall similarity in gene expression patterns (Figure 1). This suggested that the pre-B and neuroblastoma cell lines clustered to the same half of the expression tree even though THP-1 is a hematopoetic cell line. It also appeared as if while the two pre-B cell lines were rather similar the two neuroblastoma lines displayed larger discrepancies in overall expression patterns. It should be noted that the SK-N-BE(2)c cell line carries an amplified *N-myc *gene, which in the clinic is correlated to adverse outcome of the disease, while the SH-SY5Y cell line carry an *N-myc *gene in germ line configuration [18].

**Figure 2:**
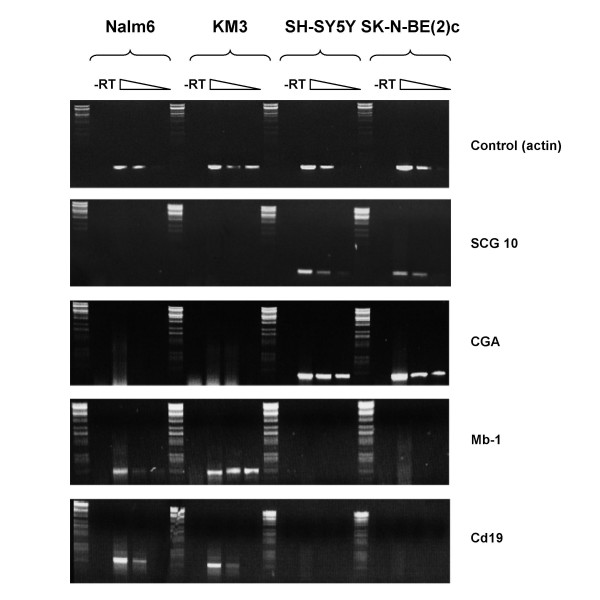
EBF target genes are expressed in a tissue specific manner. The figure show ethidium bromide stained agarose gels with the products from RT-PCR analysis of Nalm6, KM3, SH-SY5Y and SK-N-BE(2)c cells. The panel shows the expression of *actin*, *SCG 10*, *CGA*, *mb-1 *and *CD19 *mRNA as indicated. Serial dilutions using 1, 1/5 and 1/25 of the template cDNA after 25 (*actin*) and 30 (*SCG 10*, *CGA*, *mb-1 *and *CD19*) cycles of PCR were used.

The finding that these two different types of tumors did display a degree of similarity in overall gene expression patterns opened the possibility that the previously identified EBF target genes would be expressed in both cell types. To investigate this possibility we extracted RNA from pre-B cell lines as well as neuroblastoma cells and investigated the expression of the pre-B cell EBF target genes *CD19 *[19] and *mb-1 *[13] as well as the potential neuroblastoma EBF target genes *SCG10 *and *CGA *[5] by RT-PCR (Figure 2). This indicated that while the neuroblastoma cells expressed *SCG10 *and *CGA *message they did not express either *CD19 *or *mb-1 *message. The opposite pattern was observed in the pre-B cell lines where *CD19 *and *mb-1 *but not *SCG10 *or *CGA *message could be detected. This show that even though neuroblastoma and pre-B lymphoma cells to some extent share gene expression patterns, they do not appear to share the expression of specific EBF target genes.

### Neuroblastoma and pre-B lymphoma cells share the expression of the transcription factor Pax-5

**Figure 3:**
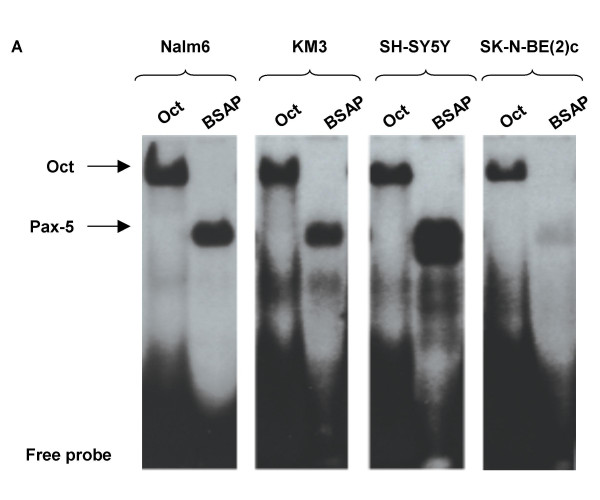
Neuroblastoma cells express constitutive levels of Pax-5 proteins.  Panel (A) display EMSAs where Oct or BSAP (Pax-5) binding sites was  incubated with nuclear extracts from Nalm6 (pre-B), KM3 (melanoma), SH-SY5Y  (neuroblastoma) and SK-N-BE(2)c (neuroblastoma) cells.

**Figure 4:**
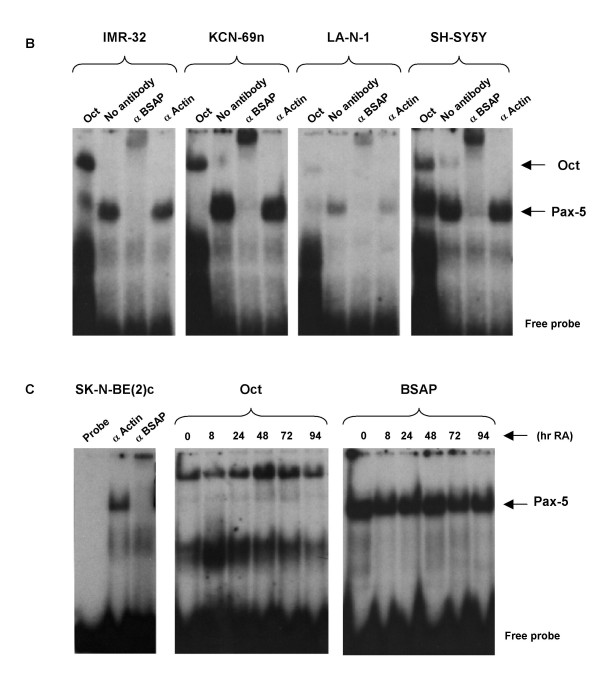
Neuroblastoma cells express constitutive levels of Pax-5 proteins.  Panel (B) shows EMSA analysis of Pax-5 (BSAP) super-shift using nuclear  extracts from a set of neuroblastoma cell lines as indicated after addition  of either no, anti-Pax-5 or anti actin antibody. Panel (C) shows auto  radiogram with EMSAs using either a Pax-5 or an Oct binding site and nuclear  extracts from SK-N-BE(2)c (neuroblastoma) cells stimulated to undergo  differentiation with retinoic acid (RA) for the indicated times. The left  panel displays a super-shift experiment using either actin or Pax-5  antisera.

Previous work has shown that both the *mb-1 *and *CD19 *genes are genetic targets for both EBF and the paired domain protein Pax-5 (BSAP) [13,19-21]. Thus, one possibility to explain lineage-restricted expression of these genes could be selective expression of Pax-5 in the Pre-B cells. To investigate this possibility we performed EMSA analysis with nuclear extracts from two human pre-B cell lines (Nalm6 and KM3) and two neuroblastoma cell lines (SH-SY5Y and SK-BE(2)c) (Figure 3A). As probes we used a consensus Oct binding site and the Pax-5 binding site from the human *CD19 *promoter [20]. These experiments revealed that not only the pre-B cell lines, but also the neuroblastoma cell lines expressed proteins able to interact with the *CD19 *Pax-5 binding site. No such binding activity was detected in extracts from epithelial cells (Data not shown). To verify that the binding activity was due to the presence of Pax-5 we performed a super-shift experiment using a Pax-5 specific antibody, a control antibody or no antibody in the binding reaction (Figure 4). While the control antibody did not affect formation of the complex the Pax-5 antibody resulted in a reduced DNA binding and also the appearance of a weak super-shifted band using either pre-B (Data not shown) or neuroblastoma nuclear extracts. These data confirmed that the DNA/protein complex observed in extracts from the neuroblastoma cells was composed of Pax-5 protein. Pax-5 protein could also be detected in the neuroblastoma cell lines IMR-32, KCN-69n and LA-N-1 (Figure 4). Since the binding activity of several transcriptions factors such as E-proteins and EBF appear to be modulated during the induced differentiation of neuroblastoma cells [5,22-24] we wanted to investigate if this was the case also for Pax-5. To this end, SK-BE(2)c cells were treated with retinoic acid and proteins were extracted at 2, 8, 24, 48, 72 and 96 hours after stimulation. The induction of differentiation was assayed by morphological change and dendrite outgrowth of the stimulated cells. We then analyzed the amount of Oct protein as well as of Pax-5 proteins at the different time points after stimulation by EMSA (Figure 4). This indicated that by using the octamer binding activity as a reference there were no major alterations in Pax-5 DNA binding in the course of SK-BE(2)c differentiation. Thus, several neuroblastoma cell lines express both EBF proteins and Pax-5, two genes known to induce expression of the *mb-1 *and *CD19 *genes in pre-B cells, but neuroblastoma cells nevertheless fail to express these two target genes.

### Pre-B lymphoma and neuroblastoma cell lines display differential expression of E-, Ets and ATF proteins

**Figure 5:**
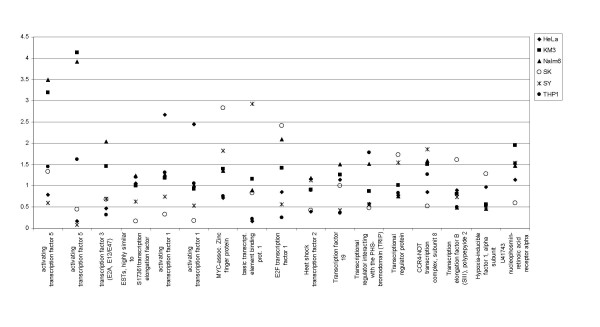
Neuroblastoma cells and lymphoma cells display specific differences in transcription factor expression. The diagram shows the  average expression levels of a set of transcription factors in the cell  lines analyzed by cDNA micro-arrays. The relative expression levels are  based on the comparison to a pooled human reference RNA.

In addition to provide information of overall relationships between different types of tumors, the micro-array analysis also yield preliminary information about differential gene expression patterns of individual genes (Appendix A). The data obtained in our experiments suggested differential expression of genes such as myb, CBFβ and HMG 1, 3 and 4 proteins with high expression restricted to the pre-B cells while Bcl-6 appeared to be over-expressed specifically in neuroblastoma cells (Appendix A). To further investigate other potential differences and similarities between pre-B and neuroblastoma cells with regard to expression of gene regulatory proteins we extracted expression data regarding additional genes encoding transcription factors from our data set (Figure 4). This analysis indicated that even though the expression of several factors appeared similar, there were large discrepancies in the mRNA expression of the E-protein E47 and the ATF5 protein. These genes were expressed to a high level in lymphoma cells but to a lower level in the neuroblastoma cells. To investigate this further we analyzed the expression of E-proteins by EMSA using a binding site from the mouse Immunoglobulin heavy chain intron enhancer as probe. This suggested that while one prominent complex was formed in the pre-B cell lines, the SK-N-BE(2)c cells did not contain any significant amounts of μE5 binding activity (Figure 5A). Several complexes were formed using nuclear extracts from SH-SY5Y neuroblastoma cells, but the migration in the gel was different from that of the complexes observed in pre-B cells. Investigating the μE5 binding activity in additional neuroblastoma cell lines suggested that nuclear extracts from IMR-32 cells gave rise to a rapidly migrating complex while extract from KCN-69n cells contained proteins forming two different complexes. This suggests that there might exist differences in E-protein expression in different neuroblastoma cell lines.

To further investigate the E-box binding activities in SH-SY5Y cells as compared to Nalm6 cells we performed super-shift assays using antibodies against E2-2, E47 or c-myc (Figure 5B). The major complexes formed by nuclear extracts from the pre-B cell lines could be super-shifted either by the addition of anti E2-2 or anti E47 antibody while the complex formed in SH-SY5Y extracts reacted only on the addition of anti E2-2 antisera. None of the complexes in any of the cell lines were affected by the addition of either the c-myc or the actin antibody. This indicates that even though E2-2 is a major component in the formation of μE5 binding activity in the pre-B cells, it appears to form complex together with E47. This was not seen in the SH-SY5Y cells suggesting that there exist a distinct difference in the composition of E-box binding complexes in the two cell types.

**Figure 6:**
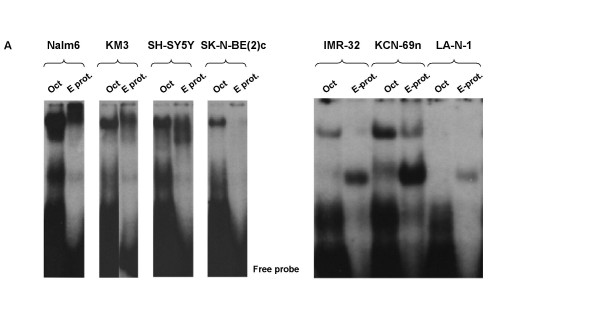
Neuroblastoma cells express variable levels of E-protein activity.  Panel (A) display auto radiograms with EMSAs of Oct and ?E-5 (E-protein) DNA  binding activity in nuclear extracts from Nalm6 and KM3 pre-B lymphoma cells  and SH-SY5Y, SK-N-BE(2)c, IMR-32, KCN-69n and LA-N-1 neuroblastoma cells.

The micro-array analysis did also suggest a difference in the expression of activator of transcription 5 (ATF5/ATF7/ATFX) [25,26]. ATF5 is a transcription factor of the basic-leucinezipper family that has been suggested to modulate neurogenesis [27] making it potentially interesting in the biology of the neuroblastoma cells. This protein binds to c-AMP responsive element (CRE) [25,26] hence, we performed EMSA analysis using oligonucleotide with a CRE binding site and nuclear extracts from either pre-B cell lines or neuroblastoma cells (Figure 6A). Both the pre-B cells and the neuroblastoma cells generated two major complexes (C1, C2) migrating with apparently the same mobility as compared to the Oct proteins. The relative abundance of these two complexes did however differ since the pre-B cells contained larger relative amounts of the slowly migrating complex (C1). This suggests that there are differences in the expression of CRE binding proteins in pre-B and neuroblastoma cells lines investigated.

**Figure 7:**
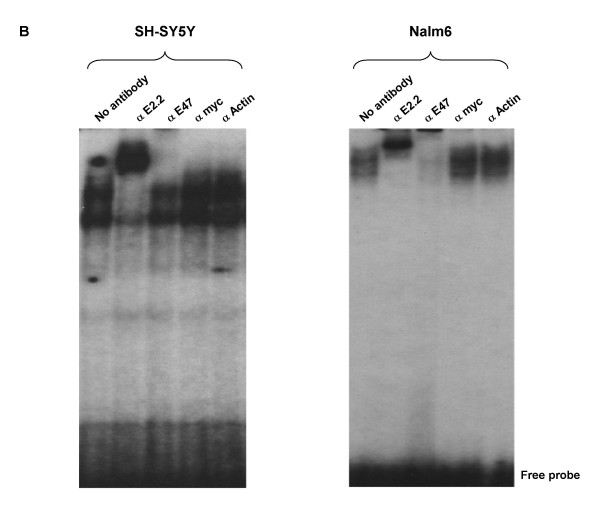
Neuroblastoma cells express variable levels of E-protein activity.  Panel (B) show a EMSA analysis where nuclear extracts from either SH-SY5Y  neuroblastoma or Nalm6 pre-B lymphoma cells were pre-incubated with either  no antibody, anti E2-2, anti E47, anti myc or anti actin antibody as  indicated.

EBF has also been suggested to share pre-B cell restricted target genes with Ets proteins and one factor belonging to this family of proteins suggested to contribute to B lineage identity is the Ets protein Pu.1 [28,29]. In order to investigate the DNA binding activities in neuroblastoma and pre-B lymphoma we incubated a PU.1 binding site (λB) from the mouse Immunoglobulin λ enhancer with nuclear extracts from the two cell types (Figure 7). This showed a complex pattern of DNA binding activities but only the pre-B cells contained a complex reacting to the addition of anti Pu.1 anti-sera. Thus, we conclude, that even though neuroblastoma and lymphoma co-express several potent transcription factors there are also specific differences. Thus, even though both cell types express EBF, the protein would have to act in a different transcription factor context in the two cell types.

### Chromatin structure participates directly in the regulation of tissue specific gene expression patterns

These experiments suggested that there indeed are specific differences in transcription factor expression between the two cell types, but also that they share expression of several key regulatory molecules. This indicates that additional components of gene regulation may be of crucial importance for target gene selection. One potential barrier to gene activation could be the chromatin structure, that is, the epigenetic setting of the cells directs the transcription factors to their tissue specific target genes. To investigate this possibility we analyzed the expression of the *mb-1 *gene in neuroblastoma cells after disruption of the chromatin structure by culturing the cells in the presence of the DNA de-methylating agent 5-azaC and the histone deacetylase inhibitor TSA. RT-PCR analysis suggested that while the untreated control cells did not express any detectable amount of *mb-1 *transcripts the 5-azaC/TSA treated cells expressed significant amounts of *mb-1 *transcripts (Figure 7). To obtain an approximation of the obtained expression levels we compared the amount of *mb-1 *message in the treated neuroblastoma cells to that of a serially diluted cDNA from Nalm6 pre-B cells. This suggested that while the expression obtained in SK-N-BE(2)c cells treated with 5-azaC/TSA was about one twentieth of that observed in a pre-B cell, the SH-SY5Y cells were induced to express levels comparable to that observed in the pre-B cells. In contrast we were unable to observe an induction of *CD19 *expression (Data not shown). Thus, we conclude that the lineage specific regulation of the *mb-1 *gene may be directly dependent on chromatin status, while expression of other pre-B cell specific EBF target genes, such as *CD19*, must be explained by other mechanisms.

## Discussion

We here report that neuroblastoma and pre-B lymphoma cells share the expression of transcription factors believed to be essential for normal B cell development while the expression of their B lineage target genes remains tissue specific. The finding that neuroblastoma cells expressed Pax-5 was somewhat surprising even though this factor has been shown to be involved in midbrain development. Mice carrying a homologous disruption of the Pax-5 gene lack both B-lymphoid cells and a fully developed central nervous system [30,31]. In the B cell compartment it also appears as if Pax-5 is crucial to lock the cell in the B-lymphoid developmental pathway. That is, even though pro-B cells from Pax-5 deficient mice express a whole set of early B cell markers [30,31], they can, in contrast to normal pro-B cells, be differentiated into other hematopoetic lineages [32,33]. Thus, Pax-5 appears to be essential for lineage fidelity in addition to be crucial for the completion of the B lymphoid development pathway. The former has been attributed to an ability of Pax-5 to inhibit the expression of cytokine receptors like M-CSF [32] and also of Notch-1 [34]. Interestingly, Notch signaling plays a key role in fate selection of neuronal cells and we have previously shown that over expression of a constitutively active form of Notch-1 inhibits induced differentiation of neuroblastoma cells [23]. It is currently not clear whether a link between Pax-5 and Notch1 expression is at hand in neuronal cells, but it is noteworthy that neuroblastoma cells express relatively high levels of Pax-5. Elevated expression levels of Pax-5 has been detected in two other tumors of neuronal origin, medulloblastoma [35] and astrocytoma [36] and *in vitro *data [37] as well as observed translocations in human B lineage tumors [38-40] indicate that Pax-5 have oncogenic properties.

In contrast to the stable expression of Pax-5 there appeared to be large differences in E-protein expression in the neuroblastoma cell lines investigated. In neuronal tissues a number of tissue specific bHLH proteins have been defined. Importantly, these pro-neuronal bHLH proteins require heterodimerization with E-proteins for the formation of DNA binding complexes. In the developing sympathetic nervous system HASH-1 is transiently expressed and is of pivotal importance for the formation of the autonomic and olfactory nervous system, neuroendocrine cells of the lung and specific regions of the telencephalon (reviewed in [41]). We have previously shown that a majority of the neuroblastomas express HASH-1, supporting the notion that the tumor is of embryonal origin [22]. We also showed that E2-2 is the preferential binding partner of the pro-neuronal bHLH protein HASH-1 in SH-SY5Y neuroblastoma cells [42], and the EMSA results presented in this paper, showing that E2-2 is the main E-protein binding a μE5-E-box, corroborate this observation. Some findings indicate that E2-2 has specific and important functions in neuronal tissues [43,44]. The gene is expressed at substantial level in brain compared to most other tissues [44] and E2-2 has been shown to bind and regulate several neuronal/neuroendocrine promoters, such as brain-specific FGF-1.B [44]. Several lines of investigation has suggested that E-proteins are redundant in B cell development and that the apparent need of E2A protein in B cell development is due to lack of sufficient doses of functional E-proteins [43,45]. This notion is substantiated by the finding that E2A deficiency can be rescued by expression of another E-protein, Heb [46], and that all these proteins appear able to activate the pre-B cell restricted λ5 promoter in synergy with EBF [47]. Thus, based on these findings it is unlikely that the E-protein composition would have any dramatic impact on EBFs ability to activate target genes in neuroblastoma.

Our data also suggest that the chromatin structure of DNA is able to modulate the function of transcription factors in a dramatic manner. In the case of the mouse *mb-1 *promoter is has been shown that methylation of specific C residues in the promoter prevents the complex formation between Pax-5 and Ets proteins, thereby reducing functional activity of the promoter [48]. Both the Ets and Pax-5 binding sites are conserved between mouse and human and similar mechanisms acting on the human promoter may well account for the observations in this report [49]. It is also noteworthy that we obtained a much higher level of *mb-1 *transcription in the SH-SY5Y cells then in the SK-N-BE(2)c cells (Figure 7), a finding that could be expected due to that the SH-SY5Y cells expressed high levels of BSAP and E-proteins while SK-N-BE(2)c cells appeared to express somewhat lower levels of BSAP and no detectable amounts of μE5 binding E-proteins (Figure 3A and 5A). However, we were unable to observe any activation of *CD19 *gene even after treatment with demethylating agents suggesting that gene regulation is exerted at several different levels or that the human CD19 gene is highly dependent also of other factors, not present in the neuroblastoma cells. In this study we disrupt chromatin structure by treating the cells with a combination of a methylation inhibitor and a HDAC inhibitor. The processes of DNA methylation and histone acetylation are intimately connected. It has been known for a long time that histone deacetylase inhibitors, by reactivating gene expression, can inhibit growth and/or survival of cancer cells. Even though the precise mechanisms behind the effect of these drugs are largely unknown several of them are now evaluated in clinical trials. Furthermore, the observations of abnormal DNA methylation patterns in malignant cells are becoming increasingly interesting, as new information of the link between gene activation and methylation status increase.

One dilemma, however, resides in the issue if a gene is demethylated due to activation or activated due to demethylation. In our specific case the latter would be the most likely explanation. We cannot however exclude that our observations are due to the induction of other unknown proteins. The selective activation of *mb-1 *and the presence of all the known crucial transcription factors in the neuroblastoma cells would, however, be in line with de-methylation being the primary event. The shared expression of a whole set of transcription factors in such diverse malignancies as lymphoma and neuroblastoma may also be of importance for our view on the relationships between different tumor types. That is, even though these tumors are highly divergent and show large differences in the clinical outcome, there may be genetic links that can be used for drug targeting thus reducing the complexity of cancer treatment dramatically.

## Conclusion

These data suggest that transcription factors are able to selectively activate target genes in different tissues and that chromatin structure plays a key role in the regulation of this activity.

## Competing interests

The author(s) declare that they have no competing interests.

## Authors' contributions

Anna Lagergren has performed the EMSAs, the micro-array experiments and the PCR experiments under the supervision of Mikael Sigvardsson while Christina Manetopoulus has performed the cell culture work as well as 5-aza-Cytidine treatments and RNA purifications under the supervision of Håkan Axelson. All contributors have been involved in the writing of the manuscript.

## Pre-publication history

The pre-publication history for this paper can be accessed here:



## Supplementary Material

Additional File 1 The data shows hierarchical clustering of the microarray data displaying differential gene expression patterns of individual genes in the different cell lines used in this study. Click here for file

## Figures and Tables

**Figure 8:**
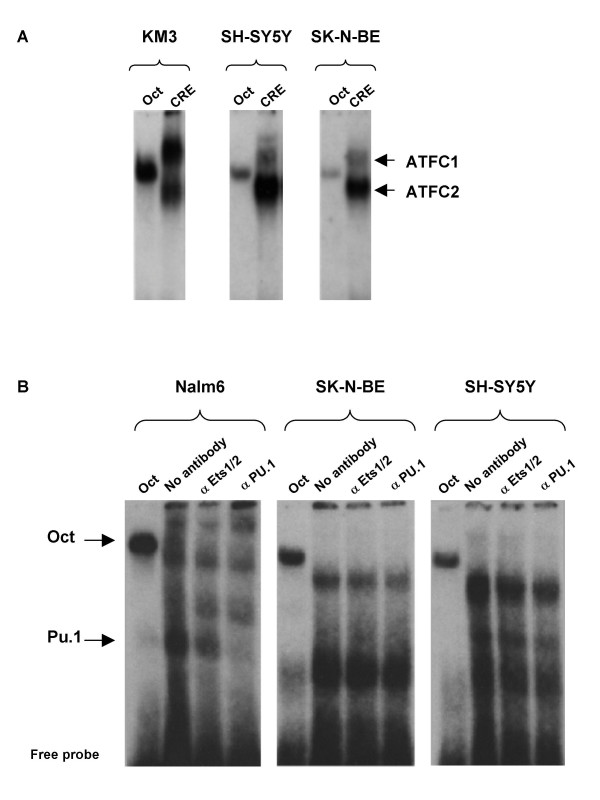
**Neuroblastoma and pre-B lymphoma differs in expression of CRE and Pu.1 site binding proteins.** Panel (A) display EMSA analysis of nuclear extracts from pre-B (KM3) or neuroblastoma cells (SH-SY5Y and SK-N-BE(2)c) incubated with a consensus cAMP responsive element (CRE). The two major complexes are denoted ATFC1 and ATFC2. Panel (B) show an EMSA analysis using nuclear extracts from either pre-B lymphoma cells (Nalm6) or the neuroblastoma cells SH-SY5Y and SK-N-BE(2)c, incubated with a labelled Pu.1 binding site from the mouse Immunoglobulin l enhancer (lB). Ets1/2 or PU.1 antibody was included in the reactions as indicated. The arrow indicates the complex super-shifted by the anti PU.1 antibody.

**Figure 9:**
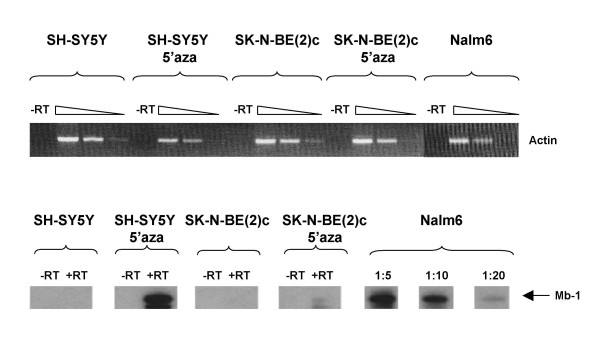
**Disruption of the chromatin structure allows for the activation of mb-1 transcription in neuroblastoma cells.** (A) The top part of the figure shows ethidium bromide stained agarose gels with actin expression after RT-PCR analysis of Nalm6, SH-SY5Y and SK-N-BE(2)c cells without or after treatment with 5-azaC and TSA. Serial dilutions using 1, 1/5 and 1/25 of the template cDNA after 25 cycles of PCR were used. Panel (B) display auto-radiograms of southern blot analysis of mb-1 RT-PCR products obtained from untreated or 5-azaC/TSA treated SH-SY5Y and SK-N-BE(2)c cells and a serial dilution of untreated Nalm6 cells after 30 cycles of PCR as indicated.
