# Supplementary material for: Neuroblastoma and pre-B lymphoma cells share expression of key transcription factors but display tissue restricted target gene expression
Source: BMC Cancer. 2004 Nov 15;4:80. doi: 10.1186/1471-2407-4-80 (PMC544580; doi:10.1186/1471-2407-4-80)

**Attachment A.**

The attachment display gene expression maps of gene sets with specific expression patterns. On top of every diagram is a hierarchical tree based on cDNA micro-array analysis of the expression of 2162 genes in a set of cell lines as indicated. Each individual hybridization experiment is represented by the name of the cell line and the number of the experiment. THP-1 is a human myeloma cell line, HeLa a human epithelial cell line, Nalm6 and KM3 represents human pre-B lymphoma. SY represents the neuroblastoma cell line SH-SY5Y and SK the neuroblasoma cell line SK-N-BE(2)c. Red represent high expression of the gene as compared to the pooled control RNA while green indicates low expression.

Scans were analyzed using GenePix Pro versions 4.0.1.9 and 4.1.1.4. BioArray Software Environment (BASE) (Saal et al. Genome Biology 2002, 3(8):software0003.1-0003.6). The settings for the analysis presented were Background Correlation: Mean FG - Mean BG, Spot filter: (Raw) SNR ch1 mean > = 2, (Raw) SNR ch2 mean > = 2, (Raw) Flags = 0, (Raw) Spot diameter > = 40, Normalization: Lowess, Reporter filter: in # of assays = 16, Analysis: Hierarchial clustering (reporter)

## Genes overexpressed in Epithelial (HeLa) cells

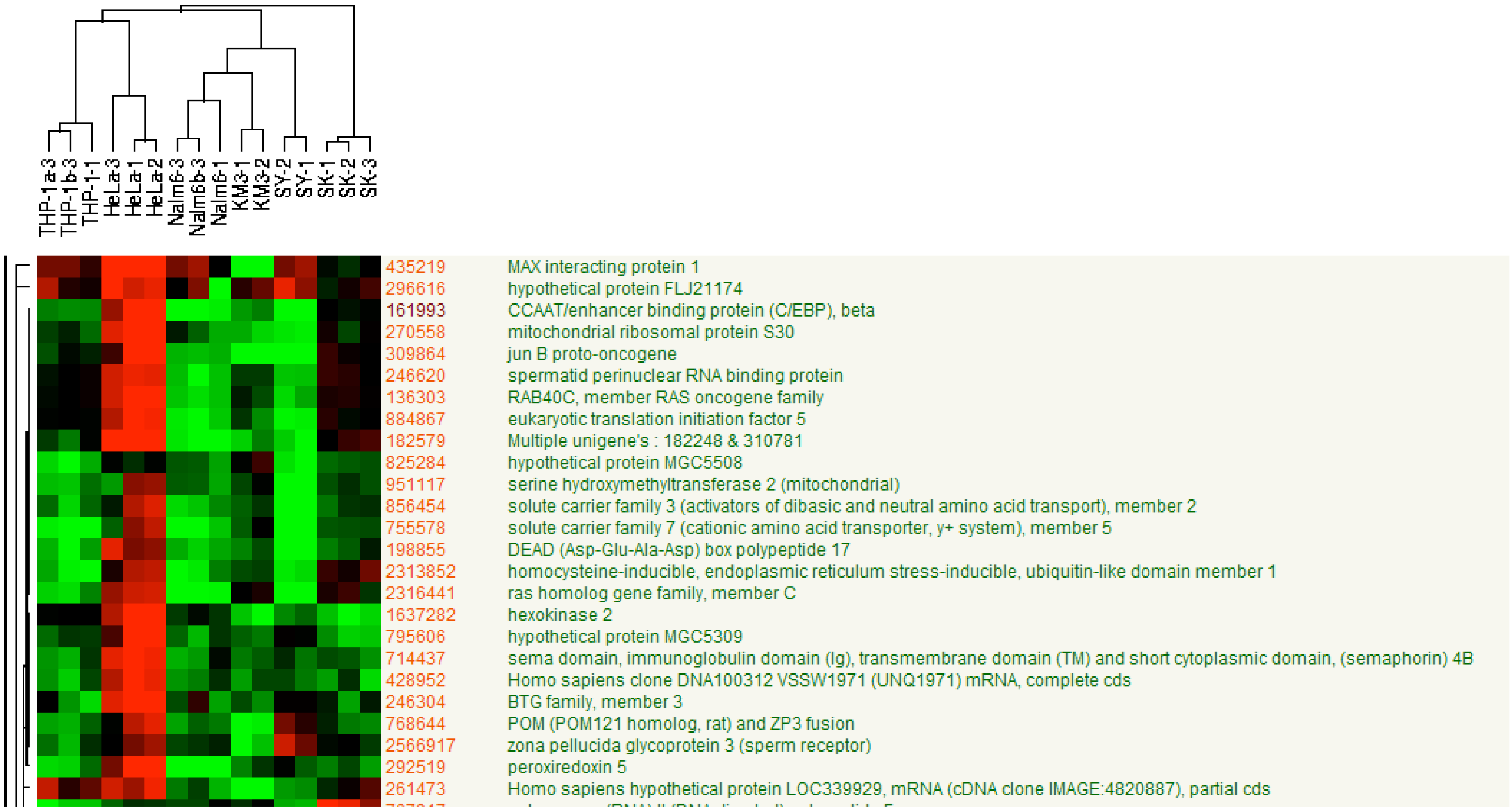

# Genes overexpressed in Epithelial and Pre-B lymphoma cells

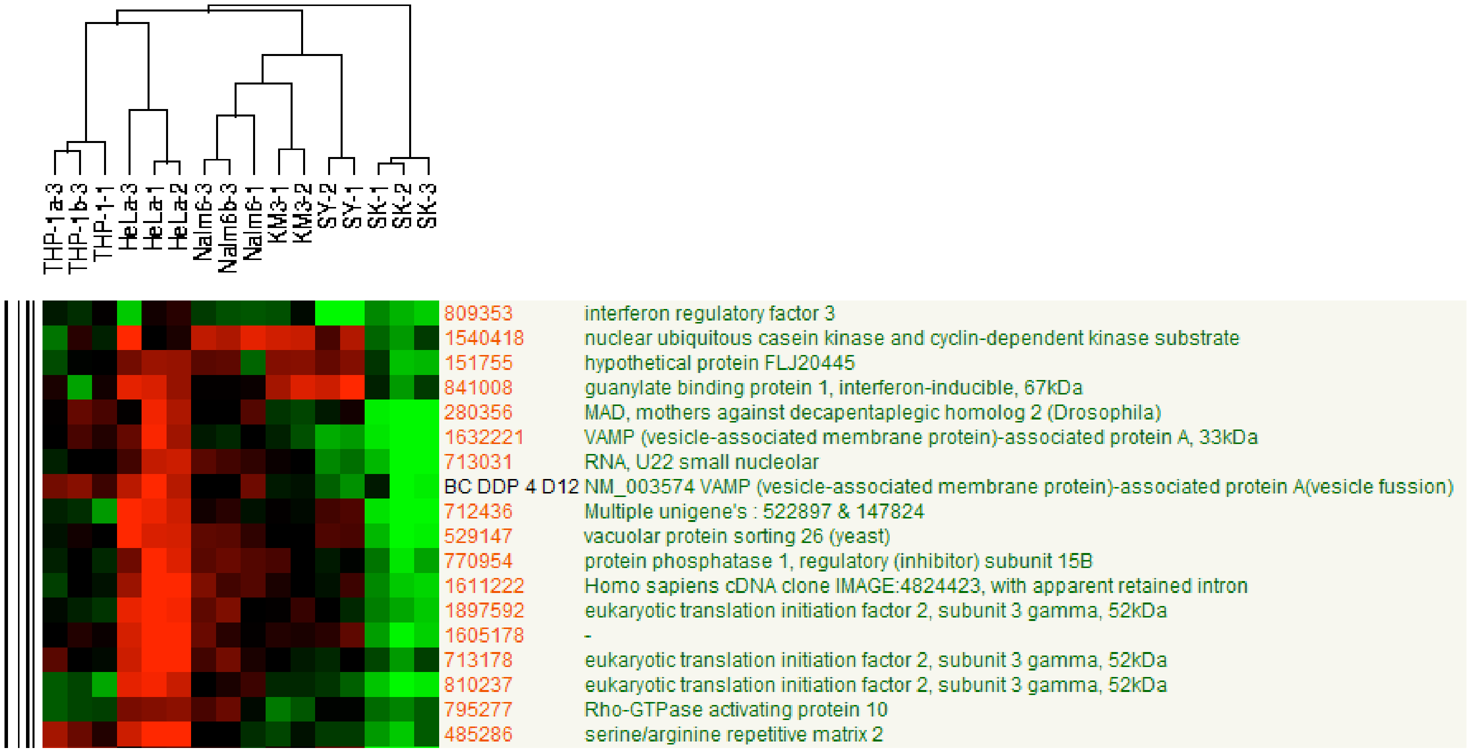

# Genes overexpressed in Neuroblastoma cells

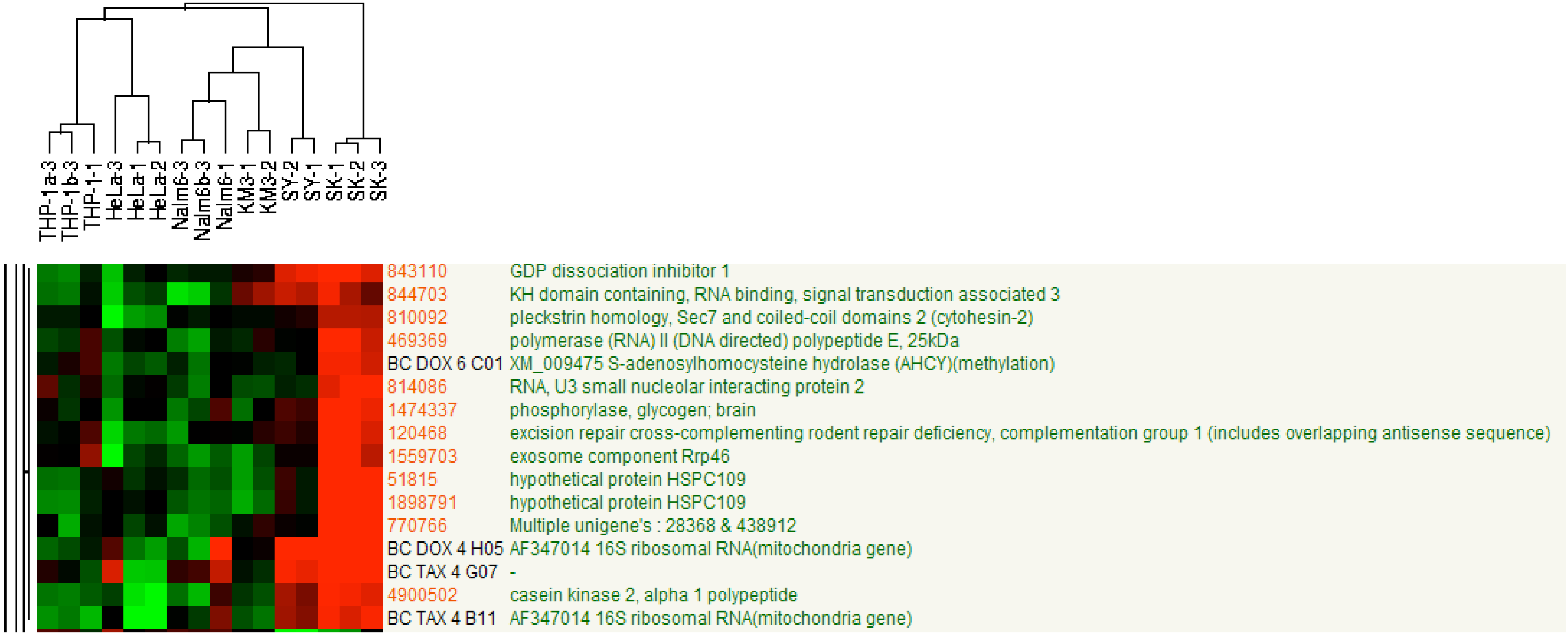

## Genes overexpressed in Myeloid leukemia and Pre-B lymphoma cells

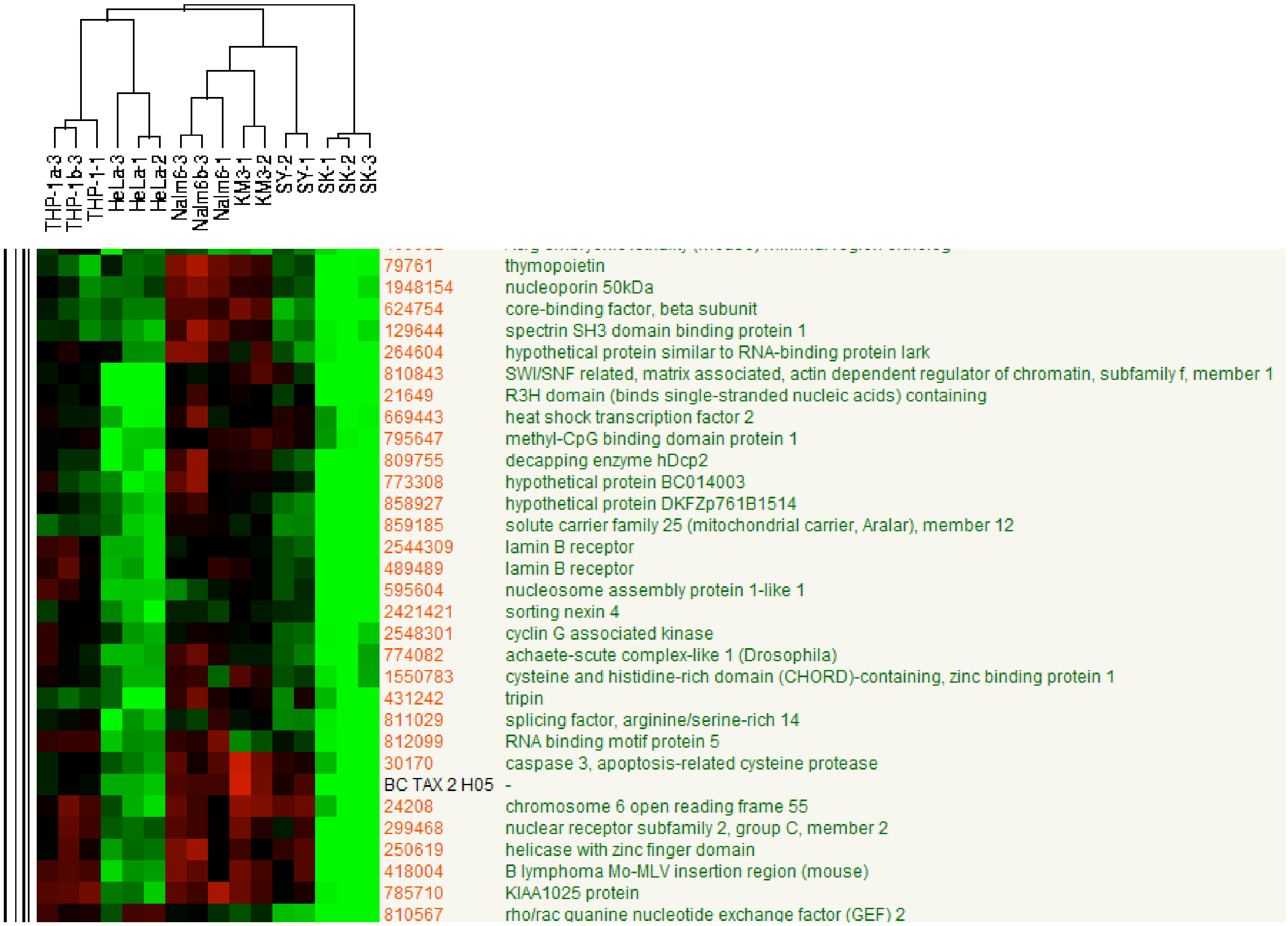

# Genes overexpressed in Myeloid leukemia cells

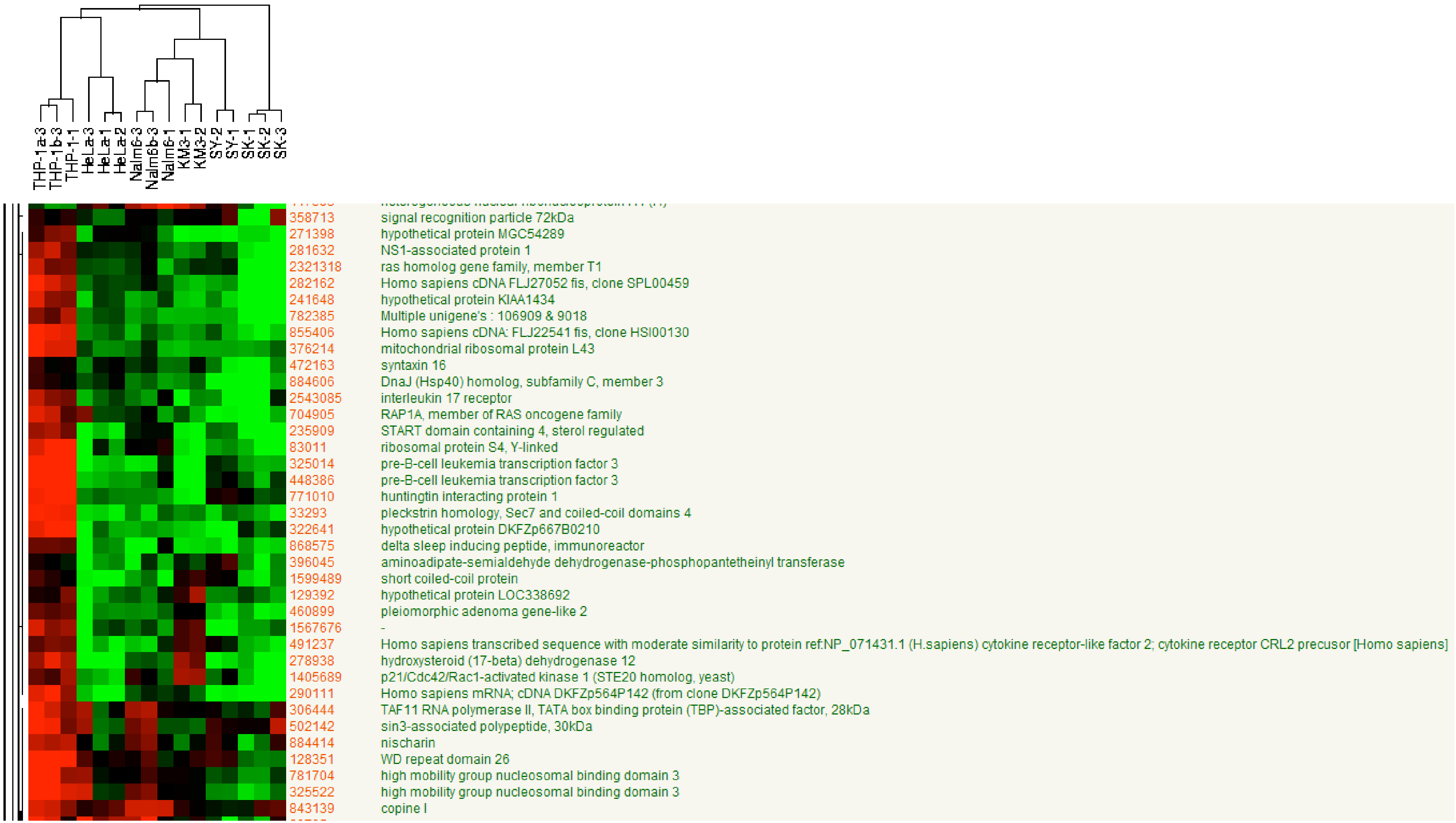

# Genes overexpressed in Neuroblastoma and Pre-B leukemia cells

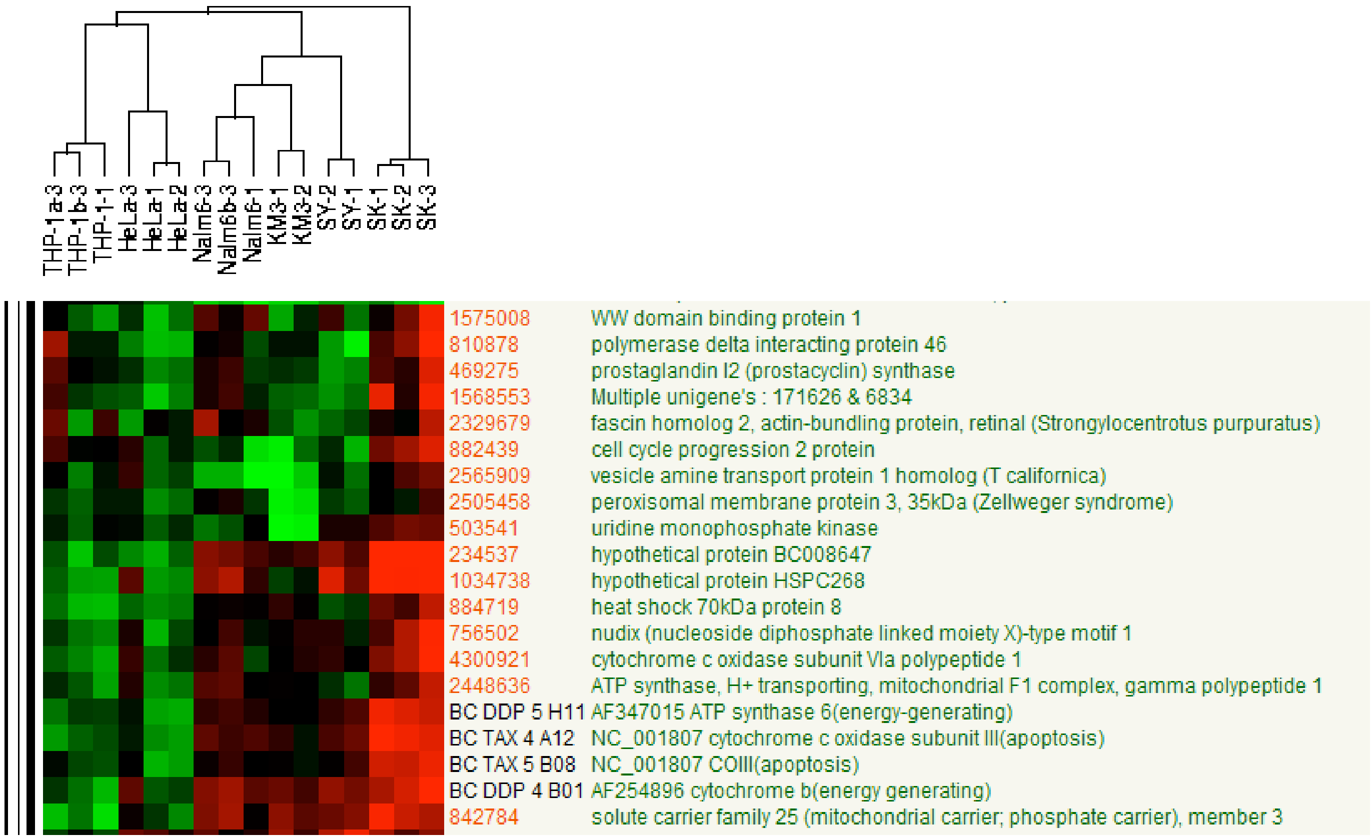

# Genes overexpressed in Neuroblastoma cells

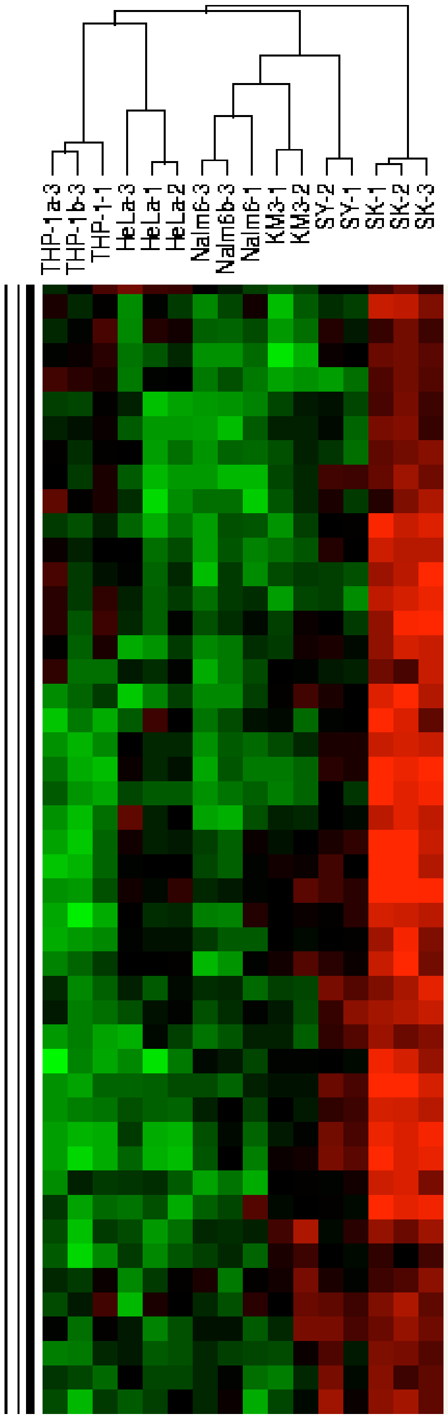


| | |
|---|---|
| [illegible] | [illegible] |
| 2314027 | mitofusin 1 |
| 853151 | ribosomal protein S16 |
| 509479 | Multiple unigene's : 448230 & 356050 |
| 897550 | hypothetical protein MGC2744 |
| BC DOX 4 A03 | - |
| 3344701 | - |
| 75644 | tenascin XB |
| 1636908 | malate dehydrogenase 2, NAD (mitochondrial) |
| 730861 | cell death-regulatory protein GRIM19 |
| 1323203 | ATPase, H+ transporting, lysosomal 56/58kDa, V1 subunit B, isoform 2 |
| 840556 | eukaryotic translation initiation factor 4E-like 3 |
| 811999 | Multiple unigene's : 77324 & 6455 |
| 811013 | adenosine monophosphate deaminase 2 (isoform L) |
| 770685 | RAB1B, member RAS oncogene family |
| 240702 | striatin, calmodulin binding protein 4 |
| 730025 | necdin-like 2 |
| 625623 | solute carrier family 5 (sodium-dependent vitamin transporter), member 6 |
| BC TAX 1 G10 | - |
| 845363 | non-metastatic cells 1, protein (NM23A) expressed in |
| BC TAX 2 C02 | NM_000269 non-metastatic cells 1 protein (NM23A) expressed in (NME1)(metastasis) |
| 884657 | translocase of inner mitochondrial membrane 8 homolog B (yeast) |
| BC DDP 3 A08 | NM_005005 NADH dehydrogenase (ubiquinone) 1 beta subcomplex 9(nuclear-encoded mitochondria gene energy-genetating) |
| 740788 | Multiple unigene's : 71962 & 165439 |
| 1947150 | solute carrier family 4, anion exchanger, member 2 (erythrocyte membrane protein band 3-like 1) |
| 323577 | solute carrier family 4, anion exchanger, member 2 (erythrocyte membrane protein band 3-like 1) |
| 824728 | microspherule protein 1 |
| 564625 | Multiple unigene's : 71787 & 325631 |
| 31842 | solute carrier family 35, member B1 |
| 788520 | Homo sapiens mRNA; cDNA DKFZp586A0722 (from clone DKFZp586A0722) |
| 563444 | GNAS complex locus |
| 1471828 | B-cell CLL/lymphoma 6 (zinc finger protein 51) |
| 545125 | nuclear transport factor 2 |
| 811097 | nucleolar protein family A, member 2 (H/ACA small nucleolar RNPs) |
| 587595 | interleukin 22 receptor, alpha 1 |
| BC DOX 6 F03 | - |
| 213176 | - |
| 384081 | etoposide induced 2.4 mRNA |
| 1636257 | dynactin 3 (p22) |
| 739230 | LIM domain binding 1 |
| 280776 | hypothetical protein MGC5139 |
| 300474 | adaptor-related protein complex 1, gamma 1 subunit |
| 770709 | Est1p-like protein B |
| 1947890 | - |
| 1859037 | eukaryotic translation initiation factor 2B, subunit 4 delta, 67kDa |
| 205303 | sorcin |
| 2018527 | dolichyl-phosphate mannosyltransferase polypeptide 3 |

## Genes overexpressed in Neuroblastoma (SK) and Epithelial (HeLA) cells

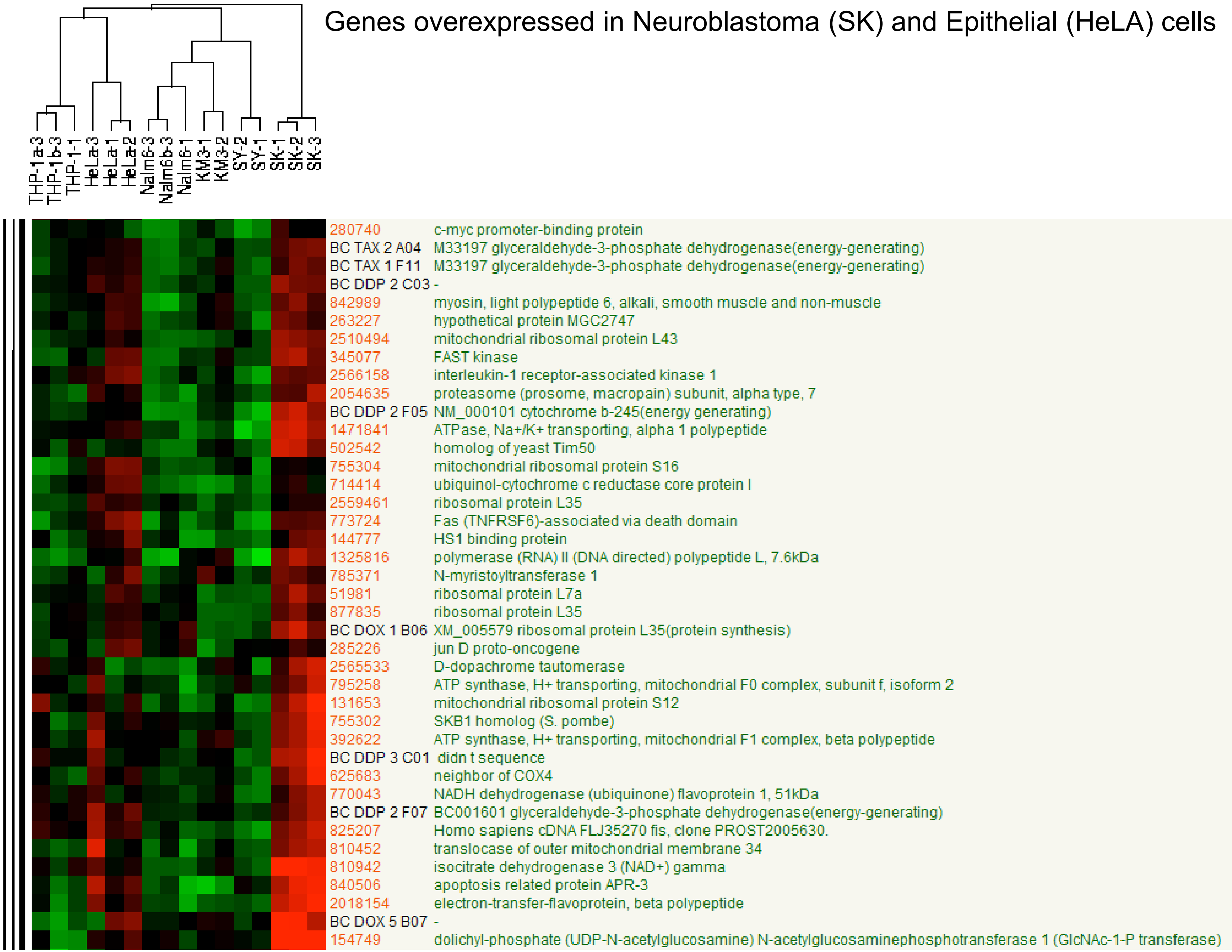

# Genes overexpressed in Pre-B lymphoma cells

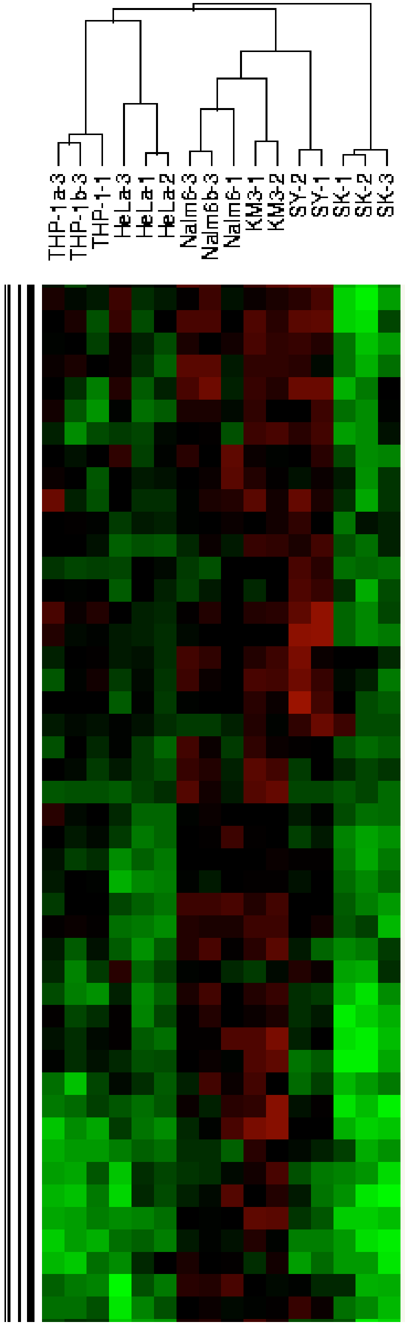


| | |
|---|---|
| 343737 | transcription factor 4 |
| 843094 | ubiquitin-like 1 (sentrin) |
| 31866 | Multiple unigene's : 77897 & 502532 |
| 273546 | phosphoribosylaminoimidazole carboxylase, phosphoribosylaminoimidazole succinocarboxamide synthetase |
| 839682 | ubiquitin-conjugating enzyme E2N (UBC13 homolog, yeast) |
| 784830 | chromosome 10 open reading frame 7 |
| 815683 | bromodomain adjacent to zinc finger domain, 1B |
| 31873 | high mobility group nucleosomal binding domain 4 |
| 810383 | Ewing sarcoma breakpoint region 1 |
| 79782 | zinc finger protein 161 |
| 282320 | BCL2-like 13 (apoptosis facilitator) |
| 758365 | CTD (carboxy-terminal domain, RNA polymerase II, polypeptide A) small phosphatase 2 |
| 131621 | hypothetical protein MGC15396 |
| 795529 | syntaxin 6 |
| 810948 | thyroid hormone receptor-associated protein, 240 kDa subunit |
| 239958 | POM121 membrane glycoprotein (rat) |
| 854746 | cell division cycle 42 (GTP binding protein, 25kDa) |
| 365531 | cell division cycle 42 (GTP binding protein, 25kDa) |
| 320146 | hypothetical protein FLJ10726 |
| 949932 | Homo sapiens similar to DNA-binding protein (LOC375708), mRNA |
| 2563366 | phosphoribosylaminoimidazole carboxylase, phosphoribosylaminoimidazole succinocarboxamide synthetase |
| 2577023 | heterogeneous nuclear ribonucleoprotein C (C1/C2) |
| 263846 | protein phosphatase 2 (formerly 2A), regulatory subunit A (PR 65), beta isoform |
| 136744 | speckle-type POZ protein |
| 1636248 | START domain containing 7 |
| 323074 | high-mobility group protein 2-like 1 |
| 240208 | - |
| 26443 | START domain containing 7 |
| 161950 | chromosome 5 open reading frame 7 |
| 815255 | caspase 2, apoptosis-related cysteine protease (neural precursor cell expressed, developmentally down-regulated 2) |
| 884892 | makorin, ring finger protein, 1 |
| 1632161 | small nuclear ribonucleoprotein D3 polypeptide 18kDa |
| 430179 | thyroid hormone receptor-associated protein, 150 kDa subunit |
| 1950727 | - |
| 341051 | pinin, desmosome associated protein |
| 784744 | M-phase phosphoprotein 6 |
| 202919 | Multiple unigene's : 507983 & 380857 |
| 825411 | heterogeneous nuclear ribonucleoprotein M |
| 322794 | pantothenate kinase 2 (Hallervorden-Spatz syndrome) |
| 1950136 | - |
| 344049 | host cell factor C1 (VP16-accessory protein) |
| 2315147 | H2A histone family, member Z |
| 2420316 | M-phase phosphoprotein 6 |
| 741841 | Homo sapiens similar to DEAD/H (Asp-Glu-Ala-Asp/His) box polypeptide 11; DEAD/H box-11 (CHL1-related helicase gene-1); yeast CHL1 homolog (LOC338812), mRNA |
| 454459 | hypothetical protein MGC40195 |
| 1417825 | transformation/transcription domain-associated protein |

# Genes down regulated in SK Neuroblastoma cells

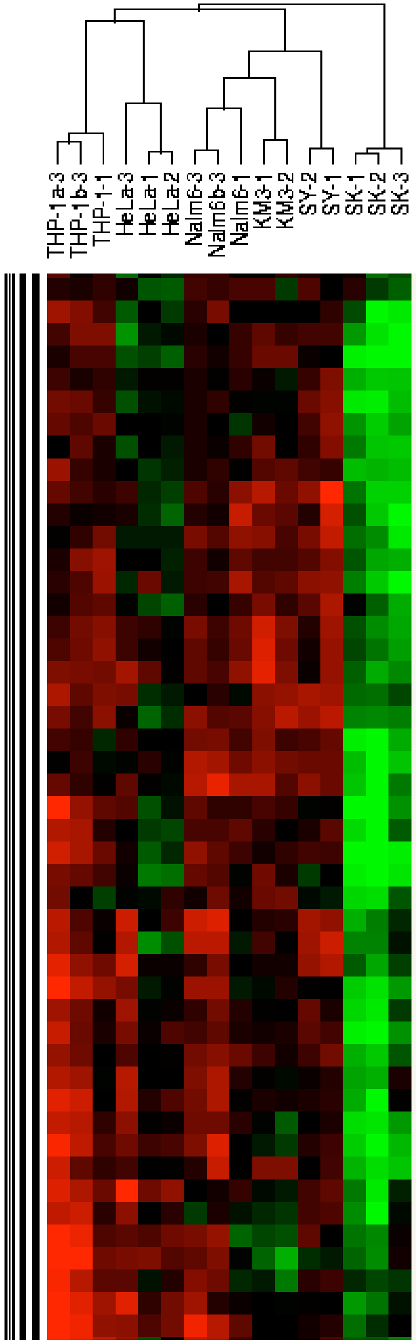


| | |
|---|---|
| 486544 | small nuclear RNA activating complex, polypeptide 3, 50kDa |
| 345525 | general transcription factor IIH, polypeptide 2, 44kDa |
| 725104 | RNA binding motif protein 16 |
| 233365 | HP1-BP74 |
| BC DDP 5 D03 | (unknown) |
| 26711 | nuclear cap binding protein subunit 2, 20kDa |
| 1535328 | karyopherin (importin) beta 3 |
| 295551 | Multiple unigene's : 435795 & 149353 |
| 247367 | NADH dehydrogenase (ubiquinone) 1 beta subcomplex, 2, 8kDa |
| 268778 | protein kinase, cAMP-dependent, regulatory, type I, alpha (tissue specific extinguisher 1) |
| 626531 | NS1-associated protein 1 |
| 869182 | CCR4-NOT transcription complex, subunit 8 |
| 745503 | zinc finger protein 9 (a cellular retroviral nucleic acid binding protein) |
| 2028057 | Homo sapiens similar to ARF GTPase-activating protein (LOC143158), mRNA |
| 340558 | actin related protein 2/3 complex, subunit 5, 16kDa |
| BC DDP 1 F05 | U41743 nucleophosmin-retinoic acid receptor alpha fusion protein NPM-RAR short form mRNA(oncogene transcriptional activity) |
| BC DDP 2 B10 | U41743 nucleophosmin-retinoic acid rec(oncogene transcriptional activity) |
| BC DOX 6 F06 | NM_002520 nucleophosmin (nucleolar phosphoprotein B23 numatrin)(RNA processing) |
| 878640 | high-mobility group box 3 |
| 322154 | Homo sapiens mRNA; cDNA DKFZp313O1721 (from clone DKFZp313O1721) |
| 32257 | Multiple unigene's : 500522 & 511425 |
| 731343 | serine racemase |
| 511586 | heterogeneous nuclear ribonucleoprotein A1 |
| 264127 | HP1-BP74 |
| 897823 | heterogeneous nuclear ribonucleoprotein D-like |
| 272632 | nuclear receptor subfamily 2, group C, member 2 |
| 1637343 | protein phosphatase 1, catalytic subunit, gamma isoform |
| 293820 | transmembrane protein 1 |
| 342211 | hypothetical protein BC016005 |
| 471859 | MADS box transcription enhancer factor 2, polypeptide D (myocyte enhancer factor 2D) |
| 272748 | Homo sapiens similar to UNR protein (LOC345463), mRNA |
| 856135 | SFRS protein kinase 1 |
| 713205 | comparative gene identification transcript 94 |
| 1519341 | KIAA0907 protein |
| 950574 | H3 histone, family 3B (H3.3B) |
| 134363 | kinesin heavy chain member 2 |
| 884525 | KIAA1041 protein |
| BC DOX 4 E06 | AF077615 TGF beta inducible nuclear protein TINP1(unknown) |
| 136933 | Homo sapiens transcribed sequences |
| 27548 | nucleoporin 153kDa |
| 359723 | mitochondrial elongation factor G1 |
| 839623 | Multiple unigene's : 69855 & 260523 |
| 131566 | hypothetical protein MGC11061 |
| 1055201 | ribosomal protein S24 |
| 417803 | peptidase (mitochondrial processing) beta |
| 884666 | DKFZP564M182 protein |
| 358752 | cystinosis, nephropathic |

# Genes overexpressed in Pre-B lymphoma cells

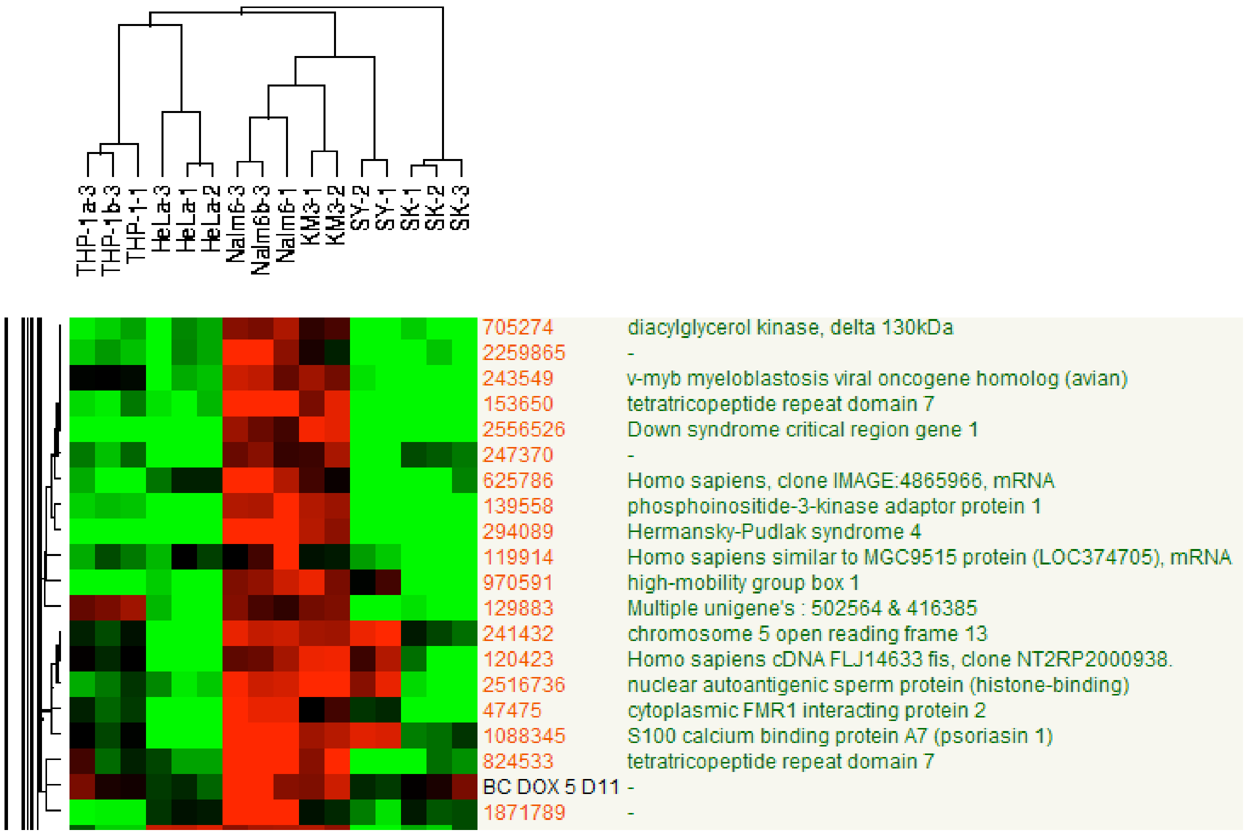

Supplement: Additional File 1 — The data shows hierarchical clustering of the microarray data displaying differential gene expression patterns of individual genes in the different cell lines used in this study. [file 1471-2407-4-80-S1.pdf]
